# Communicating With Patients Who Prefer a Language Other than English: A Curriculum on Interpreter Use for Medical Students

**DOI:** 10.15766/mep_2374-8265.11572

**Published:** 2026-01-23

**Authors:** Carolina Gonzalez Bravo, Sofia Ramirez, Kristen E. Sandgren, Amy L. Conrad, Anna Schmitz

**Affiliations:** 1 Fourth-Year Medical Student, University of Iowa Roy J. and Lucille A. Carver College of Medicine; 2 Pediatrics Clerkship Coordinator, Department of Pediatrics, University of Iowa Roy J. and Lucille A. Carver College of Medicine; 3 Clinical Associate Professor of Pediatrics and Pediatrics Clerkship Director, Division of Hospital Medicine, Department of Pediatrics, University of Iowa Roy J. and Lucille A. Carver College of Medicine; 4 Associate Professor, Division of Psychology, Department of Pediatrics, University of Iowa Roy J. and Lucille A. Carver College of Medicine; 5 Clinical Associate Professor of Pediatrics, Division of Hospital Medicine, Department of Pediatrics, University of Iowa Roy J. and Lucille A. Carver College of Medicine

**Keywords:** Pediatrics, Medical Interpreter, Flipped Classroom, Simulation, Spanish

## Abstract

**Introduction:**

Patients who prefer a language other than English (LOE) often face significant barriers in health care. Although national mandates require access to professional medical interpreters (MIs), studies indicate that interpreter services are frequently underutilized or used inappropriately. Moreover, many medical schools lack a formal curriculum dedicated to training students on the effective use of MIs.

**Methods:**

An educational curriculum was developed for second- and third-year medical students to enhance effective communication using MIs to interact with patients who prefer an LOE. The curriculum included an online module (26 minutes) followed by an in-person workshop (60 minutes) featuring simulated patient encounters with an MI. Pre- and postcourse surveys were administered to assess self-reported knowledge and comfort. Descriptive statistics and the Mann-Whitney *U* test were used to characterize responses and analyze differences between pre- and postcourse responses.

**Results:**

Over 1 year, 131 medical students participated in the curriculum. The precourse survey was completed by all 131 students, and the postcourse survey by 92 students. Scores on all survey items significantly changed, indicating more frequent or confident behaviors related to interpreter use. Postcourse satisfaction was high, with 90% of respondents reporting they were *satisfied* or *very satisfied* with the curriculum.

**Discussion:**

This educational innovation successfully addressed a critical gap in medical training by introducing a structured curriculum focused on best practices for working with MIs. The hybrid approach of an online module followed by in-person practice was a time- and resource-efficient way to integrate this information into a busy medical student schedule.

## Educational Objectives

By the end of this activity, learners will be able to:

1. Evaluate clinical situations to determine when the use of a professional medical interpreter is necessary.
2. Describe best practices for working with medical interpreters and communication techniques for patients who prefer a language other than English.
3. Recognize the role of medical interpreters as essential members of the health care team and their value in patient care.
4. Reflect on how the use or misuse of interpreters can impact patient safety, communication, and health equity.
5. Demonstrate appropriate communication strategies with a medical interpreter during an in-person workshop.

## Introduction

Patients who prefer a language other than English (LOE) and those who use American Sign Language (ASL) face significant communication barriers in health care, often resulting in reduced quality of care, increased medical errors, and poorer health outcomes.^1–3^ Despite national mandates requiring access to professional medical interpreters (MIs), these services are frequently underutilized or misused.^4–6^ A key contributing factor is the lack of formal curriculum in medical education providing training on how and when to work effectively with interpreters.^7,8^ Consequently, many learners enter clinical settings unprepared to navigate language-discordant encounters, further exacerbating disparities in care. Evidence suggests that targeted training can increase the use of interpreter services.^9^

All medical trainees can benefit from interpreter services education, ideally before starting clinical rotations and residency. Even for learners with experience working with interpreters, there are unique considerations in the clinical setting, as well as ethical and legal obligations. Students may not fully understand or appreciate the important role MIs have in patient care that goes beyond literal interpretation of what is spoken during an encounter. To address this gap, we developed an interpreter training curriculum for medical students.

To create the MI curriculum, the team appraised existing resources, many of which addressed cultural competency and the broader importance of language access. A review of *MedEdPORTAL* and the broader literature revealed publications on interpreter training for residents and medical students. However, existing resources did not meet our desire to have in-person practice while still working within our resource constraints (e.g., time, space, and money). In addition, we wanted to ensure that our curriculum covered best practices for communication strategies and effective counseling styles such as teach-back, and highlighted the enhanced cultural competency provided by MIs. Other *MedEdPORTAL* publications described the use of standardized patients and OSCEs for residents and medical students as an effective and well-received approach.^10–13^ These OSCEs require substantial time, space, and personnel resources for effective implementation. Additionally, because OSCEs are often used for medical student evaluation, students may perceive them as stressful rather than educational experiences and may not have time for open discussion and debriefing. Online modules have demonstrated success in teaching medical students how to effectively work with interpreters.^9,14^ In reviewing existing resources, we found publicly available online videos that highlight best practices when working with interpreters.^15–19^ There were videos for working with ASL interpreters and others from medical schools specifically related to working with MIs; however, we did not find a video that covered everything we deemed important such as describing and showing best practice techniques in the clinical setting, providing a brief history of medical interpretation, as well as special considerations for ASL.

Other publications described effective in-person sessions, including training on telephone interpretation.^20,21^ There were robust programs and curriculums for medical trainees, though most were designed to last longer than 1 hour or take place over multiple sessions.^17,22,23^ Aside from the simulation activity described by Mundo and colleagues, which included ASL interpreters, few other resources were found for this unique population.^21^ To our knowledge, no existing curriculum combines a video module with a brief (1-hour) skills-based workshop, specifically designed for medical students, focusing on the practical use of interpreters, including ASL interpreters.

## Methods

We developed a curriculum at the University of Iowa Carver College of Medicine with a 26-minute online video and a 1-hour workshop to teach communication skills for patients whose preferred language is an LOE, including ASL. It was designed for second- and third-year medical students in their pediatrics clerkship. There was no space in the preclinical curriculum for additional material; we therefore integrated this topic into an existing didactic session within the pediatric clerkship, which all medical students complete during their core clinical year. Students start their core clinical year in January of their second year of medical school; therefore, many students completed this curriculum prior to starting their third year, and all completed it prior to their fourth year. This ensured all graduating medical students would receive standardized education on and practice with MIs. No formal training in the use of MIs had been provided prior to or after this point in the medical school curriculum. Session participants included a clerkship faculty member, a facilitator, 2 bilingual volunteers, and 2 MIs. Facilitators for the in-person workshop included faculty members and graduate medical trainees (fellows and residents), all of whom had experience working with medical students and MIs. Two volunteers who were bilingual in Spanish and English were needed for each session, and volunteers ranged from medical students and residents to hospital and medical school staff. Due to the need for multiple bilingual volunteers and limited access to ASL and non-Spanish interpreters, the in-person sessions were unable to facilitate practice with languages other than Spanish and English.

Each facilitator received a guide that included background information, session logistics, suggested debriefing prompts, and an overview of the educational objectives (Appendix A). The MIs were certified and employed by our institution. The facilitators, MIs, and volunteers did not receive any formal training; they were provided with the written material, including the facilitator guide, and a brief (5-minute) overview at the beginning of the session by a clerkship faculty member, who was present for each session to provide oversight and answer questions.

### Curriculum

Learners were instructed to complete a video module (Appendix B) on interpreter use before the workshop. The video was based on an outline developed by a fourth-year medical student, hospitalist, MI, and education specialist. The outline was derived from a review of published videos and modules that addressed working with MIs, including ASL interpreters.^15–20^ The resources ranged from organizations such as the National Association of the Deaf to publicly available MI education videos from other medical schools, such as the Stanford University School of Medicine.^15–20^ Through compiling resources and publications, along with cross-referencing videos, we sought to include pertinent information on best practices for working with MIs, focusing on information relevant to medical students. In addition, expert opinion and input from the institution's lead MI were incorporated. Learners were provided with a link to the video, which was viewable on a secure institutional website and allowed students to watch at their own pace. The video module was typically completed within 1 to 2 weeks prior to the in-person session, at the students’ convenience. There was no tracking system in place to ensure that students watched the video. The video link included the precourse survey (Appendix C), with instructions to complete both; reminders were sent prior to the in-person session.

The in-person component took place during a designated 60-minute session within the clerkship schedule, comprising approximately 20 to 30 students per session. The sessions followed a pair-and-share approach, with role-playing based on clinical cases. Students were divided into smaller groups of about 10 per group. Each group included a facilitator, a bilingual volunteer acting as a caregiver, and an MI. Facilitators guided students through different simulated clinical scenarios, using a combination of role-play between the students, MI, and a Spanish-speaking caregiver.

Two cases were developed by members of the research team, clerkship faculty, and an MI to represent two common pediatric clinical cases. The cases were designed to allow students to focus on communication, counseling style, and cultural competency rather than medical knowledge. One case simulated a clinic visit that included answering common questions about home remedies for viral respiratory infections (Appendix D). The second case simulated an inpatient encounter and included providing discharge instructions (Appendix E). The students had a chance to briefly interview the caregiver followed by providing anticipatory guidance and answering the caregiver's questions. The students and facilitators focused on the interactions with the interpreter and caregiver, rather than on the content of their interview or counseling. Students practiced identifying when to use an interpreter and how to effectively engage with MIs. Students were then provided with real-time feedback from the facilitator and MIs for each case scenario.

Each scenario was followed by a group debriefing, where facilitators, MIs, and fellow students provided constructive feedback. The student groups had time to reflect on the role of MIs, their value in patient care, and how their use or misuse can affect patient safety. The facilitator and clerkship faculty member helped guide these discussions, and MIs/volunteers also provided real-world examples when indicated. The session concluded with a summarization of key learning points for effective communication with MIs.

### Pre- and Postcourse Surveys

To evaluate the impact of our curriculum, we used pre- and postcourse surveys. The surveys were online, were voluntary, and included multiple-choice questions with self-reported, Likert-scale responses. No demographic information was obtained. These self-assessments primarily evaluated learners’ reactions, perceived learning, and anticipated behaviors following the educational interventions. To gauge how well the educational objectives were met, students were asked to rate their comfort in working with MIs, as a proxy for learning and behavioral change related to interpreter use. All survey questions were developed and reviewed by the research team, and then evaluated and approved by the dean for medical student education at University of Iowa Carver College of Medicine and the local Institutional Review Board. The questions were designed to assess comfort levels before and after the learning activities; they were not validated.

A link to complete the precourse survey (Appendix C) was provided with the online module, and learners were asked to complete the precourse survey prior to the in-person learning workshop. Learners completed the postcourse survey (Appendix F) online immediately after the in-person workshop. The postcourse survey included an additional question to assess overall satisfaction with the curriculum (both video and in-person sessions) and provided space for open-ended comments and feedback. No objective measures were collected.

As responses were anonymous, pre- and postcourse responses could not be paired. Normality of pre- and postcourse responses was assessed using the Shapiro-Wilk test, and measures of skewness were calculated. Descriptive statistics and nonparametric tests (Mann-Whitney *U* test for differences between unpaired samples) were run using SPSS software version 29. Two team members analyzed narrative data, identifying and summarizing common themes.

## Results

A total of 131 students participated in the in-person session of this workshop focusing on the practical use of MIs, including ASL interpreters, in clinical practice. All 131 students completed the precourse survey; 92 completed the postcourse survey. We were unable to track how many students watched the video module. The link for the survey and video was the same, so presumably a similar number of students watched the video and completed the survey. None of the responses followed a normal distribution (Shapiro-Wilk test of normality, range .85 to .90 [precourse] and .60 to .84 [postcourse], *p* < .001). Responses on three postcourse survey questions were significantly skewed, including those gauging the learners’ frequency of assessing need for an MI (*M* = 2.36 [*SE* = 0.25]) and frequency of use of an MI (*M* = 1.24, [*SE* = 0.25) (both rated on a 5-point Likert scale, where 1 = *always*, 5 = *never*), and their comfort with using proper techniques for working with an MI (*M* = 2.36 [*SE* = 0.25]) and frequency of use of an MI (*M* = 1.24 [*SE* = 0.25]; both rated on a 5-point Likert scale, where 1 = *always*, 5 = *never*), and their comfort with using proper techniques for working with an MI (*M* = 1.03 [*SE* = 0.25]; rated on a 5-point Likert scale, where 1 = *very comfortable*, 5 = *not comfortable at all*). Most responses were at the lower end of the scale, indicating more frequency and comfort in using MIs.

We therefore analyzed shifts in central tendency and distribution of responses using nonparametric tests for unpaired samples (Mann-Whitney *U* test). The question related to comfort in using MIs during patient encounters yielded responses on the precourse survey showing that, overall, students felt somewhat comfortable using MIs (*M* = 2.82 [*SD* = 0.95]) and somewhat comfortable with using interpreter techniques (*M* = 2.76 [*SD* = 0.87]), and felt slightly less comfortable with using ASL interpreters (*M* = 2.98 [*SD* = 0.96]; all rated on a 5-point Likert scale, where 1 = *very comfortable*, 5 = *not comfortable at all*). All scores significantly improved on the postcourse survey, indicating greater comfort levels after participation in the course (*p* < .001 vs precourse).

**Figure 1.**
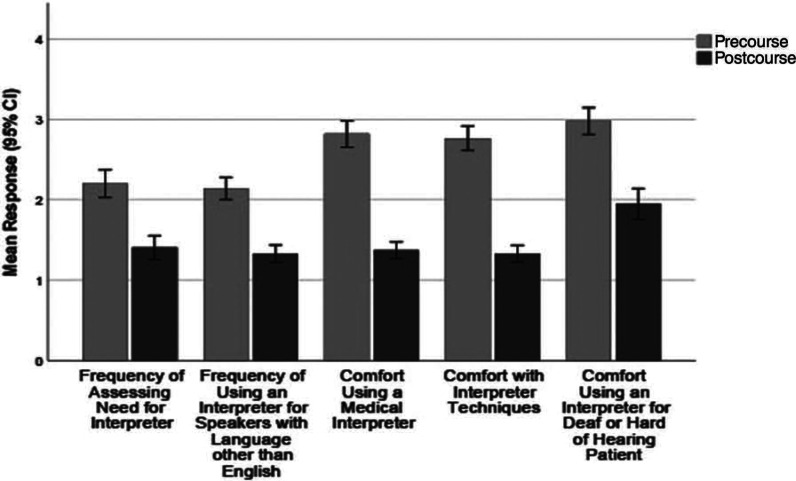
Pre- and postcourse survey responses (*N* = 131 and *N* = 92, respectively) related to frequency of assessing the need for a medical interpreter (MI) and frequency of use of an MI (rated on a 5-point Likert scale; 1 = *always*, 5 = *never*) as well as responses related to comfort working with MIs and interpreter techniques (each rated on a 5-point Likert scale; 1 = *very comfortable*, 5 = *not comfortable at all*). All pre/post comparisons were significantly different (*p* < .001 by Mann-Whitney *U* test). Values are mean ratings with 95% CI.

For the two questions related to frequency of assessing the need for an MI and using MIs (each assessed with a 5-point Likert scale, where 1 = *always*, 5 = *never*), students reported on the precourse survey that they usually assessed the need for an MI (*M* = 2.2 [*SD* = 0.99]) and usually used MIs (*M* = 2.14 [*SD* = 0.79]). The postcourse survey showed a significant improvement in both of these behaviors after course participation (each *p* < .001 vs precourse). Details are shown in Figure 1.

**Figure 2.**
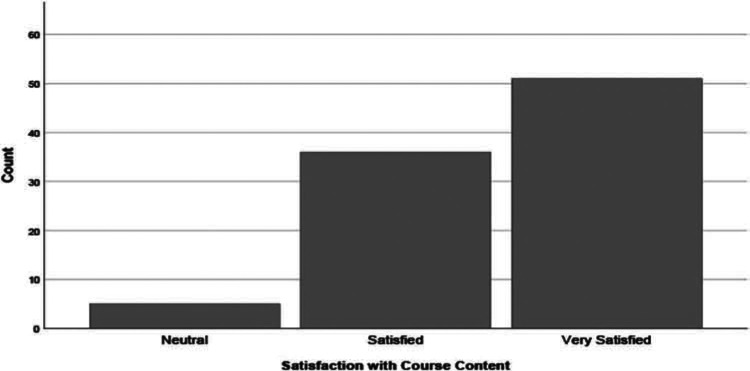
Postcourse responses on the survey question gauging learners’ self-reported satisfaction with the curriculum (*N* = 92), rated on a 5-point Likert scale (1 = *very satisfied*, 5 = *not satisfied at all*). No respondents indicated satisfaction levels of *not satisfied* (score of 4) or *not satisfied at all* (score of 5).

The majority of students reported high levels of satisfaction with the curriculum; 90% of the 92 respondents on the postcourse survey indicated that they were either *satisfied* or *very satisfied* with the curriculum (Figure 2).

Fifty students provided open-ended feedback, with the majority of responses being positive. Many highlighted the value of deliberate practice in a safe and supportive setting. One student shared, “It was beneficial to have experience working with an interpreter. It allowed us to practice, ask questions, and get more comfortable with these interactions.” Another noted, “I think it was good to have real practice and feedback from both the patient and the interpreter.” Several students suggested that this curriculum would be most helpful if introduced prior to the start of clinical clerkships. The only neutral or negative feedback came from a student who already had experience with interpreters and felt the session was less relevant. Suggestions for future improvement included incorporating practice with patients who are Hard of Hearing or blind, to further expand training across diverse communication needs.

## Discussion

This educational innovation successfully addressed a critical gap in medical training by introducing a structured curriculum focused on best practices for working with MIs. The success of video modules and the valuable experience of hands-on simulated practice were combined to create a hybrid approach. Aspects of published OSCEs were transformed into a less resourced, smaller-budget, and lower-stakes (no formal evaluation) session to still achieve simulated practice. This curriculum represents a novel contribution to the literature, offering a flexible and scalable approach that can be easily integrated into a busy medical school schedule. The curriculum was well-received by learners, with high satisfaction ratings and qualitative feedback emphasizing the value of deliberate practice, real-time feedback, and exposure to realistic clinical scenarios. Pre- and postcourse surveys revealed significant improvements in self-reported knowledge, comfort, and behaviors related to interpreter use, suggesting that the intervention was effective in promoting more confident and appropriate use of MIs in clinical care.

The development, implementation, and evaluation of our interpreter curriculum offered several key lessons that may be valuable to other educators seeking to improve communication training with patients who prefer an LOE. One of the primary challenges we faced was finding space within an already full medical school curriculum. As a result, the material was presented early on in clinical rotations but not before. Additionally, we were limited in both the time and structure available for interpreter-specific education. While we would have preferred a longer or longitudinal format, we adapted by employing a blended learning approach that combined an asynchronous video module with a focused in-person workshop.

Developing the online module presented both benefits and challenges. We found no existing resource that fully addressed the content and approach we envisioned, necessitating the creation of a custom module. Although the development and production of the module required significant up-front investment in time and resources, the finished product provided a consistent and reusable tool that reduced demands on in-person teaching time. However, we recognize that not all institutions may have access to the technical or financial resources required for high-quality video production.

In contrast, the in-person workshop, while requiring less initial development, depended heavily on human resources. Facilitators, MIs, and volunteers who spoke Spanish were all crucial to the session's success. Due to institutional policies, we are unable to compensate volunteers. For institutions without these human resources, a similar in-person practice session with video and phone interpreters that are more readily available may be a viable option. Despite these logistical constraints, our evaluation data and the structure of the in-person session suggest that the curriculum met the educational objectives.

Despite the overall success of our initiative, several limitations should be considered when evaluating the outcomes and generalizability of our work. First, the study was conducted at a single institution with second- and third-year medical students. This limited the diversity of participants and may affect the generalizability of the results to other students in health care settings. We did not obtain demographic information, including prior experience with interpreters, or any unique identifiers. Therefore, we were unable to match pre- and postcourse surveys for individual students. As a result, we were unable to measure the extent to which each student's scores changed, which could have provided a more detailed understanding of the learning process and the specific impact of the intervention on individual students. This limitation also meant that we were unable to track long-term knowledge retention and actual utilization of the material over time. We did not have a mechanism to track how many students completed the video prior to the in-person session, so it is possible that some learners missed valuable background information. Due to the availability of volunteers and MIs, we were able to conduct in-person practice with Spanish-speaking interpreters only. In future sessions, we would like to expand to include other languages, including ASL.

The evaluation approach, relying on self-reported surveys, has its own limitations. While self-assessments can provide valuable insights into students’ perceptions of their knowledge and comfort levels, they are inherently subjective and may not capture actual behavioral changes in clinical settings. Furthermore, the absence of objective measures, such as direct observations of student behavior or performance, limits our ability to definitively assess the transfer of skills to clinical practice. The feedback from students was largely positive, but the responses were self-reported and may be subject to response bias, as students who were satisfied with the curriculum may have been more motivated to provide feedback. This could lead to an overrepresentation of positive comments and an underrepresentation of constructive criticism.

In response to student comments, proposed future iterations of this curriculum include introduction in preclinical years and incorporating more languages, including ASL, in the practice sessions. Going forward, it would be beneficial to implement more robust evaluation methods, such as OSCEs or direct observation of students in clinical settings, to provide a more comprehensive assessment of the training's effectiveness. Other objective measurements to track over time could include hours of interpreter use (in-person and/or by phone) and documentation of interpreter use in the medical record. Another important future step is to expand this curriculum beyond medical students to include other health care learners such as nursing, physical therapy, physician assistant, and pharmacy students. Interprofessional education in this area would foster collaborative practice and ensure that all members of the health care team are equipped with the skills and awareness necessary to use MIs effectively in clinical care.

From a broader perspective, our work supports the need for formal, structured training in MI use to be embedded as a standard component of medical education. Our findings reinforce the value of experiential learning and the importance of normalizing interpreter use as a core skill in providing equitable and high-quality care. Educational policymakers and curriculum committees may consider using blended learning models—combining online modules with in-person simulations—as a scalable and resource-efficient way to integrate this content across institutions.

## Appendices


Facilitator Guide.docxBridging the Language Gap Video Module.mp4Precourse Survey.docxInterpreter Module 1 Clinical Scenario.docxInterpreter Module 2 Clinical Scenario.docxPostcourse Survey.docx
*All appendices are peer reviewed as integral parts of the Original Publication.*
