# Supplementary material for: Communicating With Patients Who Prefer a Language Other than English: A Curriculum on Interpreter Use for Medical Students
Source: MedEdPORTAL. 2026 Jan 23;22:11572. doi: 10.15766/mep_2374-8265.11572 (PMC12827796; doi:10.15766/mep_2374-8265.11572)
Supplement: Supplementary file 1 — Facilitator Guide.docxBridging the Language Gap Video Module.mp4Precourse Survey.docxInterpreter Module 1 Clinical Scenario.docxInterpreter Module 2 Clinical Scenario.docxPostcourse Survey.docx [file mep_2374-8265.11572-s001.zip › D. Interpreter Module 1 Clinical Scenario.docx]

**STUDENT**

**A**

During this activity, you will work as a group to utilize an in-person interpreter in a simulated clinic encounter. Read the patient information and goals below, and then take turns with your group members obtaining the information outlined below. The goal of this activity is to practice your knowledge of appropriate interpreter use. You will not be evaluated on the content of your interviewing skills during this activity.

**HPI**

You are in Gen Peds Clinic seeing Sylvia Ramirez, a 6-month-old female infant who comes in for an illness with a cough. The clinic medical assistant who checked in your patient noted that the family speaks Spanish and requested a Spanish-speaking interpreter during the visit.

Goal: Utilize the in-person interpreter to obtain a history from Sylvia’s caregiver about her illness. You may consider asking questions regarding the following:

- Timing of the illness
- Symptoms
- Feeding/Input
- Diapers/Output
- Medical History
- Surgical History
- Family Medical History
- Social History
- Allergies

**A**

- Immunizations

**STUDENT**

**B**

During this activity, you will work as a group to utilize an in-person interpreter in a simulated clinic encounter. Read the patient information and goals below, and then take turns with your group members obtaining the information outlined below. The goal of this activity is to practice your knowledge of appropriate interpreter use. You will not be evaluated on the content of your interviewing skills during this activity.

| **Counseling and Recommendations** | |
| --- | --- |
| You diagnose the baby with a viral URI.  **Goal:** Using the guide below, counsel the family on your recommendations for management | |
| Diagnosis   - Explain to the caregiver that you are diagnosing an upper respiratory tract infection caused by a virus.   Prognosis/Progression   - Most babies do very well with viral infections, and it can be expected to pass within a week.   - The cough can last a couple weeks.   - It is not uncommon for babies under 1 year of age to get viral illnesses every few weeks. - Keep watching baby’s symptoms for worsening. - Antibiotics are not helpful for viral infections.   Dehydration   - Keep the baby hydrated, offer feeds frequently.   - If baby has only 1-2 diapers in a day, then recommend they come back to be evaluated. | Fever   - Fevers at this age can be expected and aren’t always worrisome.   - High fevers of 103 to 104 are not abnormal and are not harmful to children.   - Can Use Tylenol and will provide her weight-based dose.   - If fevers associated with ear pulling and increased fussiness: the baby may have an ear infection and can be brough back in.   - If fevers persist for more than 3-4 days would recommend bringing the baby back in to be seen.   Respiratory Distress   - Watch for increased work of breathing.   - If baby seems to be breathing very fast and pulling in at the ribs would recommend being re-evaluated at the clinic or the ER. - You can use a humidifier at nighttime to help with the cough. - Honey is not recommended for cough at this age due to risk of Botulism. |

**A**

**CAREGIVER**

In this activity, the students have been asked to utilize the in-person interpreter to help with obtaining a history for a clinic patient that you as the caregiver brought in. Information for the history of this patient has been provided for your use below. The goal of the activity is for the students to practice their skills utilizing interpreters and they should not be evaluated on the content of their interviewing skills. Once the activity is done, please provide feedback to the students on your experience as the caregiver.

**A**

| **HPI** | |
| --- | --- |
| You are the parent of a 6-month-old Sylvia who has been sick. | |
| - How long:   - It started a couple days ago. - Symptoms:   - She has been really congested and has a bad cough, especially at nighttime.   - Has had one fever up to 101F.   - No difficulty breathing. - Feeding:   - Baby is bottle fed but doesn’t seem very interested in feeding. No vomiting, no diarrhea. - Diapers:   - Still having the normal amount of wet and dirty diapers. - Medical History:   - Born at 38 weeks. No issues with delivery. No medical Issues. No Medications. | - Surgical History:   - None - Family Medical History:   - No important family medical history. Everyone is healthy. - Social History:   - Attends Daycare. First baby. - Allergies:   - None - Immunizations:   - Up to date |

**CAREGIVER**

**B**

In this activity, the students have been asked to utilize the in-person interpreter to help with providing counseling for a clinic patient that you as the caregiver brought in. Examples of questions you might ask as a caregiver have been provided for your use below. The goal of the activity is for the students to practice their skills utilizing interpreters and they should not be evaluated on the content of their counseling. Once the activity is done, please provide feedback to the students on your experience as the caregiver.

**Counseling and Recommendations Questions:**

Feel free to ask some of these questions during the counseling as appropriate. You are a first-time parent and are nervous about having a sick baby.

- She just got over a cold, is it normal for her to be sick this often?
- I feel like she isn’t feeding well, how can I be sure that she is getting enough to drink?
- Is a high fever harmful to the baby?
- I’ve never used Tylenol; how do I know how much to give her?
- Her cough seems really bad at night, and she isn’t sleeping at all. What Can I do?
- Could we give her some antibiotics to help her get over this quickly? I always feel better when my doctor gives me antibiotics.
- My Abuela always says that honey is helpful for coughs, is it safe to give her some?
