# Supplementary material for: Communicating With Patients Who Prefer a Language Other than English: A Curriculum on Interpreter Use for Medical Students
Source: MedEdPORTAL. 2026 Jan 23;22:11572. doi: 10.15766/mep_2374-8265.11572 (PMC12827796; doi:10.15766/mep_2374-8265.11572)
Supplement: Supplementary file 1 — Facilitator Guide.docxBridging the Language Gap Video Module.mp4Precourse Survey.docxInterpreter Module 1 Clinical Scenario.docxInterpreter Module 2 Clinical Scenario.docxPostcourse Survey.docx [file mep_2374-8265.11572-s001.zip › E. Interpreter Module 2 Clinical Scenario.docx]

**STUDENT**

**A**

During this activity, you will work as a group to utilize an in-person interpreter in a simulated inpatient encounter. Read the patient information and goals below, and then take turns with your group members obtaining the information outlined below. The goal of this activity is to practice your knowledge of appropriate interpreter use. You will not be evaluated on the content of your interviewing skills during this activity.

**PATIENT INFORMATION**

You are rounding in the hospital and seeing a 2-year-old with asthma who was admitted 2 days ago with an asthma exacerbation. You are talking with the caregiver to see how the child did overnight and how they are doing today. Your goal is to ask about the following:

- Overnight Issues
- Feeding
- Sleeping
- Oxygen needs
- Respiratory symptoms: coughing, increased work of breathing, etc.
- Albuterol needs
- Energy Levels/Activity

**A**

- How are parents feeling about discharge?

**STUDENT**

**B**

During this activity, you will work as a group to utilize an in-person interpreter in a simulated inpatient encounter. Read the patient information and goals below, and then take turns with your group members obtaining the information outlined below. The goal of this activity is to practice your knowledge of appropriate interpreter use. You will not be evaluated on the content of your interviewing skills during this activity.

| **Discharge Counseling:** | |
| --- | --- |
| The child has been off the oxygen for over 12 hours and last used albuterol at 8 pm last night. You think they are ready for discharge and are counseling the family on discharge. Your goal is to answer all caregiver questions and provide the following information: | |
| - The child has been off oxygen and seems to be doing well, we think they are ready for discharge. - At home- continue to monitor for fevers, worsened or continued respiratory symptoms, or poor feeding. Would recommend being return for evaluation by their primary doctor or the ER in case of worsening symptoms. - We will be sending home with a steroid called prednisolone.   - Take the steroids twice daily for the next 3 days.   - If symptoms do not improve significantly by this time, this course may need to be lengthened. | - We will also send home with Albuterol with a mask and spacer.   - The respiratory therapist can show you how to use the mask and spacer prior to discharge.   - Use the albuterol 2-6 puffs if the child has coughing fits or is working harder to breathe.   - If needing the albuterol more than 3-4 times a day or the child continues to have difficulty breathing despite the albuterol, then we recommend being evaluated. - What pharmacy would you like those sent to? - We will also create an asthma action plan to send home, so you know what to do if symptoms occur. - An order to follow up in the pulmonology clinic will be placed.   - You should hear from them in about a week.   - In your chart we have Spanish listed as your preferred language. They should call with an interpreter, otherwise you can ask them for a Spanish interpreter, and they can get one for you. - Antibiotics are not helpful for viral infections or asthma. |

**A**

**CAREGIVER**

In this activity, the students have been asked to utilize the in-person interpreter to help with obtaining an inpatient, overnight history for an admitted inpatient that you are the caregiver for. Information for the history of this patient has been provided for your use below. The goal of the activity is for the students to practice their skills utilizing interpreters and they should not be evaluated on the content of their interviewing skills. Once the activity is done, please provide feedback to the students on your experience as the caregiver.

**PATIENT INFORMATION**

Overnight Events

(Feel free to make up your own answer if not one of these questions asked)

*You are the parent of a 2-year-old child with asthma who has been admitted to the hospital for 2 days with an asthma exacerbation.*

**A**

| Overnight Issues   - She had a few coughing fits overnight, but less than before. - She came off the oxygen around midnight.   Feeding   - Feeding better, was able to drink her whole milk this morning and a little bit of breakfast.   Sleeping   - Still being woken up when she coughs but slept better last night.   Oxygen needs   - She pulled the oxygen off herself around midnight and was able to stay off for the rest of the night. | Respiratory symptoms: coughing, increased work of breathing, etc.   - Continues to have the cough intermittently but improved. - She doesn’t seem to be working hard to breath anymore. - Has a lot of nasal congestion and drainage still.   Medications:   - Used the albuterol around 8 pm last night for some breathing issues and coughing fit but has required any since.   Energy Levels/Activity   - Seems more like herself and is asking to go home.   How are parents feeling about discharge?   - Ready to go home, but nervous because her symptoms always seem to be worse at night. |
| --- | --- |

**B**

**CAREGIVER**

In this activity, the students have been asked to utilize the in-person interpreter to help with providing counseling an inpatient ready for discharge to you as the caregiver. Examples of questions you might ask as a caregiver have been provided for your use below. The goal of the activity is for the students to practice their skills utilizing interpreters and they should not be evaluated on the content of their counseling. Once the activity is done, please provide feedback to the students on your experience as the caregiver.

**Counseling and Recommendations Questions:**

Feel free to ask some of these questions during the discharge counseling as appropriate. This is the first time the child has been hospitalized with asthma.

- What caused her asthma and why does she need the steroids?
- What do I do if she starts looking bad again at home?
- We have only ever used a nebulizer for albuterol, we have never used a spacer. How will I know how and when to use it?
- She goes to preschool; how can I tell the teachers what to do if she has problems there?
- I have had difficulty with setting up appointments in the past because they always call me and speak in English. How do I get her visit with the lung doctor set up?
- Could we give her some antibiotics to help her get over this quickly? I always feel better when my doctor gives me antibiotics.
