# Supplementary material for: Communicating With Patients Who Prefer a Language Other than English: A Curriculum on Interpreter Use for Medical Students
Source: MedEdPORTAL. 2026 Jan 23;22:11572. doi: 10.15766/mep_2374-8265.11572 (PMC12827796; doi:10.15766/mep_2374-8265.11572)
Supplement: Supplementary file 1 — Facilitator Guide.docxBridging the Language Gap Video Module.mp4Precourse Survey.docxInterpreter Module 1 Clinical Scenario.docxInterpreter Module 2 Clinical Scenario.docxPostcourse Survey.docx [file mep_2374-8265.11572-s001.zip › A. Facilitator Guide.docx]

**Facilitator Guide: Medical Interpreter Session**

**Audience**: Medical Students during core pediatric clerkship
**Duration**: 60 minutes
**Format**: Two groups, each assigned one module (Clinic Visit or Inpatient Admission)

**Background:** Prior to this session, students were given access to a video module to provide baseline information on how to work with a medical interpreter. After watching the video and by the end of this activity, learners will be able to:

1. Evaluate clinical situations to determine when the use of a professional medical interpreter is necessary.

2. Describe best practices for working with medical interpreters and communication techniques for patients who prefer a language other than English

3. Recognize the role of medical interpreters as essential members of the healthcare team and their value in patient care.

4. Reflect on how the use or misuse of interpreters can impact patient safety, communication, and health equity.

5. Demonstrate appropriate communication strategies with a medical interpreter during an in-person workshop.

**Key learning points for verbal communication:**

- Establish rapport with the interpreter before seeing the patient.
- Explain your role to the patient and allow the interpreter to introduce themselves.
- Speak directly to the patient in a normal tone of voice.
- Use pauses between sentences and ask one question at a time.
- Use first-person language when addressing the patient.

**Session Breakdown (60 Minutes Total)**

| Segment | Time | Activity | Roles |
| --- | --- | --- | --- |
| 1. Welcome & Overview | 10 min | - Introduce session goals and structure - Explain importance of interpreter use in clinical care with reference to the video module - Assign groups to Module #1 or #2 | Facilitator |
| 2. Group Activity: History Taking | 15 min | - Students interview caregiver using interpreter - Use provided patient history (Module #1: sick infant; Module #2: asthma admission) | Students, Interpreter, Caregiver (role-play), Facilitator (observer) |
| 3. Group Activity: Counseling & Recommendations | 15 min | - Students provide counseling and answer caregiver questions - Use provided question prompts | Students, Interpreter, Caregiver, Facilitator |
| 4. Group Debrief & Feedback | 15 min | - Each group shares reflections - Caregiver and interpreter provide feedback - Discuss challenges and best practices  - Reflect on how the use or misuse of interpreters can impact patient safety, communication, and health equity | Facilitator leads; all participants contribute |
| 5. Wrap-Up & Key Takeaways | 5 min | - Summarize learning points - Reinforce respectful and effective interpreter use - Thank participants | Facilitator |
