# Supplementary material for: Communicating With Patients Who Prefer a Language Other than English: A Curriculum on Interpreter Use for Medical Students
Source: MedEdPORTAL. 2026 Jan 23;22:11572. doi: 10.15766/mep_2374-8265.11572 (PMC12827796; doi:10.15766/mep_2374-8265.11572)
Supplement: Supplementary file 1 — Facilitator Guide.docxBridging the Language Gap Video Module.mp4Precourse Survey.docxInterpreter Module 1 Clinical Scenario.docxInterpreter Module 2 Clinical Scenario.docxPostcourse Survey.docx [file mep_2374-8265.11572-s001.zip › C. Precourse Survey.docx]

Pre-Course Survey

1. How often do you assess the need for a medical interpreter with patients who might need one?
   1. Always
   2. Most of the time
   3. Sometimes
   4. Rarely
   5. Never
2. How often do you use a medical interpreter with a patient who has limited English proficiency?
   1. Always
   2. Most of the time
   3. Sometimes
   4. Rarely
   5. Never
3. How comfortable do you feel using a medical interpreter during patient encounters?
   1. Very comfortable
   2. Somewhat comfortable
   3. Neither comfortable nor uncomfortable (Neutral)
   4. Somewhat uncomfortable
   5. Not comfortable at all
4. How comfortable are you with the proper techniques for working with a medical interpreter (positioning, where to look, how to ask things, etc.)?
   1. Very comfortable
   2. Somewhat comfortable
   3. Neither comfortable nor uncomfortable (Neutral)
   4. Somewhat uncomfortable
   5. Not comfortable at all
5. How comfortable are you using a medical interpreter when communicating with a patient that is deaf or hard of hearing?
   1. Very comfortable
   2. Somewhat comfortable
   3. Neither comfortable nor uncomfortable (Neutral)
   4. Somewhat uncomfortable
   5. Not comfortable at all
